# Weight Outcomes of Latino Adults and Children Participating in the Y Living Program, a Family-Focused Lifestyle Intervention, San Antonio, 2012–2013

**DOI:** 10.5888/pcd12.150219

**Published:** 2015-12-10

**Authors:** Deborah Parra-Medina, Yuanyuan Liang, Zenong Yin, Laura Esparza, Louis Lopez

**Affiliations:** Author Affiliations: Yuanyuan Liang, Laura Esparza, University of Texas Health Science Center at San Antonio, San Antonio, Texas; Zenong Yin, University of Texas at San Antonio, San Antonio, Texas; Louis Lopez, YMCA of Greater San Antonio, San Antonio, Texas.

## Abstract

**Introduction:**

US Latinos have disproportionately higher rates of obesity and physical inactivity than the general US population, putting them at greater risk for chronic disease. This evaluation aimed to examine the impact of the Y Living Program (Y Living), a 12-week family-focused healthy lifestyle program, on the weight status of adult and child (aged ≥7 years) participants.

**Methods:**

In this pretest–posttest evaluation, participants attended twice-weekly group education sessions and engaged in physical activity at least 3 times per week. Primary outcome measures were body mass index ([BMI], zBMI and BMI percentile for children), weight, waist circumference, and percentage body fat. Wilcoxon signed-rank tests and mixed effects models were used to evaluate pretest–posttest differences (ie, absolute change and relative change) for adults and children separately.

**Results:**

BMI, weight, waist circumference, and percentage body fat improved significantly (both absolutely and relatively) among adults who completed the program (n = 180; all *P* ≤ .001). Conversely, child participants that completed the program (n = 72) showed no improvements. Intervention effects varied across subgroups. Among adults, women and participants who were obese at baseline had larger improvements than did children who were obese at baseline or who were in families that had an annual household income of $15,000 or more.

**Conclusion:**

Significant improvements in weight were observed among adult participants but not children. This family-focused intervention has potential to prevent excess weight gain among high-risk Latino families.

## Introduction

Latinos have disproportionately higher rates of obesity and physical inactivity than the general US population, putting them at greater risk for chronic disease. US Latino children aged 2 to 19 years have the highest obesity prevalence (22.4%) compared with non-Latino black (20.2%) and non-Latino white (14.1%) children (1). US Latino adults have the second-highest obesity prevalence (42%), following non-Latino blacks (47.8%) (1). Adult and child family members share diet and physical activity (PA) behaviors and obesity status (2). Given the connection between individual behavior and household environment, multilevel healthy lifestyle interventions targeting all household members are a promising approach to improving individual behaviors. Involving the entire family is an important cultural adaptation for interventions designed for Latinos, who are highly group-oriented and consider family the primary source of identity and social support (3).

Most intergenerational obesity interventions are child-focused and have a parent component. Family interventions typically limit participation to parent–child dyads and examine outcomes only of child participants (2). Few interventions explore the effect of incorporating the entire family, particularly when family is broadly defined as all household members, regardless of age (child or adult) and relationship (sibling, parent, grandparent). Several small studies demonstrated that programs involving all household members promote healthy eating and PA and prevent excess weight gain (4–6). However, these studies did not target racial/ethnic minorities or financially disadvantaged populations who have higher rates of obesity than the general population. Latinos are more likely than non-Latino whites to identify cultural or linguistic preferences; low health literacy; and problems relating to transportation, neighborhood safety, cost, and resource availability as barriers to adopting healthy behaviors (7,8). Little information exists on culturally targeted lifestyle interventions designed for all household members of Latino families.

This article describes Y Living, a culturally appropriate, family-focused, community-based healthy lifestyle program for Latino families and examines its effect on weight measures of adult and child participants.

## Methods

The evidence-based Y Living program, funded by the Cancer Prevention and Research Institute of Texas, was designed by the University of Texas Health Science Center at San Antonio (UTHSCSA) and the YMCA of Greater San Antonio (YMCA) to prevent excess weight gain by increasing adoption of health-promoting PA and dietary behaviors. Y Living aligns with national cancer prevention guidelines (9) and The Guide to Community Preventive Services’ recommendations for reducing obesity and improving PA (10). We used a pretest–posttest design to evalute the program. Participants enrolled between January 2012 and June 2013. This program evaluation was determined to be exempt (Category 2) from review by UTHSCSA’s institutional review board.

### Setting and recruitment

The program took place in San Antonio, Texas, an urban city in Bexar County with high rates of obesity and chronic disease. San Antonio has 1.4 million residents and a median household income of $45,722; about 20% of residents live in poverty, and 63% of residents are Latino (11). In Bexar County, 65% of residents are overweight or obese (12). The local YMCA serves more than 35,000 members with PA and health and wellness education at 8 facilities. Y Living was offered at 3 of the 8 facilities serving predominantly Latino and low-to-middle-income families.

YMCA recruited families via area churches, schools, organizational newsletters, neighborhood newspapers, and word of mouth. After completing an interest form, 1 adult family member participated in a structured screening interview with a YMCA staff member who ascertained the prospective family’s commitment to a 12-week program offered in English that required the entire family to attend twice-weekly group education sessions, engage in PA at least 3 times per week, and (family members aged ≥7 years) participate in assessments. Families not able to commit to the program received referrals to alternative YMCA programs. Families indicating commitment to sustained participation (12-week program plus 3-month maintenance) were scheduled for baseline assessment before the program’s start. Other than their time, enrolled families incurred no cost and received a 3-month YMCA family membership and program materials (eg, program binder, journal, T-shirt, pedometer). YMCA offered childcare during education sessions for very young children.

### Measurements

The program was evaluated using a pretest–posttest design. UTHSCSA researchers developed standardized data collection protocols and trained and certified YMCA staff members in data collection procedures. Data collection training addressed equipment management, privacy, safety, and assessment administration. Participants (106 children aged 7–17 y, 242 adults ≥18 y) underwent standardized assessments before and immediately following the 12-week program. Anthropometrics were repeated at weeks 4 and 8.

Participants provided demographic information as part of their YMCA member registration: age, sex, race/ethnicity, years of education, household structure (number of adults and number of children), and annual household income. Waist circumference was measured to the nearest 0.25 inch using a MyoTape Body Tape Measure with tension scale gauge. Height was measured to the nearest 0.25 inch using a fixed stadiometer without shoes or socks. A bioelectrical impedance analysis machine (Tanita SC-331S) was used to determine body fat percentage, body weight, and body mass index ([BMI] kg/m^2^). BMI percentile and zBMI for children were determined using CDC BMI-for-age growth charts (www.cdc.gov/growthcharts).

### Y Living Program

A local YMCA’s executive director and advisory board developed Y Living to address a local need for culturally appropriate opportunities in which families learn to improve health by adopting healthy lifestyle behaviors. After an initial pilot, UTHSCSA researchers were invited to collaborate with YMCA to enhance the program by integrating evidence-based behavioral strategies. Consistent with the Social Cognitive Theory of behavior change, the program emphasized self-regulation, behavioral capability, observational learning, and self-efficacy (13) to support the entire family by proactively addressing intrafamilial, sociocultural, and environmental factors (14).YMCA’s overarching goal of achieving total wellness by enriching spirit, mind, and body and local leaders’ knowledge of the community was integrated throughout the program.

**Training.** Education sessions were delivered by YMCA staff or community volunteers with professional expertise in curriculum topics (eg, registered dietitians delivered nutrition education). Staff and volunteers, most of whom were bilingual or bicultural, resided or worked in the program site’s community and were experienced with its unique geographic and sociocultural issues. YMCA staff members’ education and training included a bachelor’s degree and work experience in the health and wellness field as well as YMCA certifications (eg, Principles of Healthy Lifestyles, Foundations of Strength and Conditioning, CPR, First Aid).

Preparation of staff members included training in healthy lifestyle and disease prevention; PA and dietary guidelines; interpreting results of body composition assessments; designing and implementing safe and enjoyable PA for inactive, overweight individuals and new exercisers; using motivational interviewing techniques with participants; and implementing the Wellness Consultation curriculum. Although Y Living is not a weight-loss program, staff received weight-loss education materials to share with participants on request.

Volunteers received a program orientation and training on healthy lifestyle, chronic disease prevention, and the delivery of presentations to local intergenerational, Latino audiences. Using community volunteers with relevant content expertise (eg, dietitians, bankers, pastors) to deliver program components is consistent with YMCA’s service-delivery model that leverages local expertise, fosters community ownership of the program, and offers a sustainable implementation strategy.

**Health education. **YMCA staff members facilitated group health education sessions twice weekly (Appendix A). Each 50-minute session started with a brief sharing time, transitioned to the health education presentation (led by a staff member or volunteer), and concluded with a group reflection and a highlighting of a community resource. Participants were exposed to new knowledge (eg, dietary guidelines) and skills (eg, goal setting, self-monitoring, problem solving) to support health behavior change.

All participants participated in health education through interactive large- and small-group discussions, demonstrations, and hands-on activities. Presenters drew on the knowledge and experience of families and encouraged participants to learn from each other. Education materials and presentations were provided in English and designed to meet the needs of an intergenerational, low-literacy audience. Presenters used photographs and other images in presentations, used food models in nutrition lessons, provided recipes incorporating traditional or familiar locally available ingredients, and emphasized free or low-cost PA in the vicinity for the entire family. To maximize the engagement of children in education sessions, special children’s versions of education materials (eg, a budget worksheet for children) were developed for many sessions. Some activities required family members to collaborate, such as building a healthy plate with magazine pictures and creating a family poster depicting agreed-on family wellness goals.

**Physical activity.** YMCA staff members led 1-hour group PA sessions after each health education session. Given the emphasis on introducing participants to a variety of PA, staff members offered different activities each week. Adults and children participated together in some activities and separately in others. Popular activities included Zumba, strength training (eg, with resistance bands or body weight), group walks, and activity stations offering brief, fun, game-like activities through which small groups of participants rotate.

**Wellness consultations.** Each family received a 30- to 45-minute wellness consultation with a YMCA staff member in week 2, week 5, and week 9. Adults received a report characterizing key dietary habits reported on baseline surveys on which behavior-change efforts could focus. Staff members reviewed recent anthropometric measurements, reinforced key health education messages, and provided additional support for behavior-change strategies tailored to the needs, concerns, and priorities of individual families. Staff members and families collaboratively explored promoters of and barriers to healthy behaviors in the home and in the broader sociocultural setting. Staff members distributed relevant health education materials, discussed individual and family goals, and provided a journal and pedometer to monitor progress.

**Special events.** Staff members and volunteers accompanied families on a grocery store tour to support families’ healthy food purchasing through practical application of health education concepts. Together, participants attended a community event (eg, Síclovía, San Antonio’s open streets event) to promote social support and connection to community resources promoting a healthy lifestyle. At the program’s conclusion, families attended an all-day outdoor weekend event at a YMCA camp and a graduation ceremony and celebration.

### Analysis

Descriptive statistics were used to summarize participant characteristics at baseline for adults and children separately. The intervention effect was evaluated using the Wilcoxon signed-rank test for adults and children separately. Two types of change scores were calculated: 1) absolute change (week 12 measure minus baseline measure) and 2) relative change ([absolute change divided by baseline measure] multiplied by 100). We used a nonparametric approach because the distribution of change scores was not normal and mostly skewed (Appendix B). Characteristics of participants who had post-intervention measures (completers) were compared with those who did not (drop-outs) using the Fisher exact test for categorical variables and the Mann–Whitney U test for continuous variables. Mixed effect models were conducted to further evaluate the association between the change in outcome measures and participant baseline characteristics: sex, age, ethnicity (Latino or not Latino), family income (<$5,000; $5,000–$9,999; $10,000–$14,999; $15,000–$24,999; $25,000–$34,999; $35,000–$49,999; ≥$50,000), family structure (Group 1, families with only 1 adult and no children; Group 2, families with >1 adult and no children; and Group 3, families with ≥1 adult and ≥1 child aged <18 y), and BMI for adults and children separately, while taking into account the correlations among same-family participants. Sensitivity analyses were conducted to deal with missing data using the multiple imputation with chained equations approach (15); the results did not change, so they are not reported. All statistical tests were conducted at a 2-sided significance level of .05, and all analyses were conducted using Stata SE version 13 (StataCorp LP).

## Results

From January 2012 through June 2013, the Y Living program was delivered 11 times in 3 facilities with an average of 15.9 families per program. Among the 348 participants enrolled, 242 (69.5%) were adults and 106 (30.5%) were children (Table 1). The median age of adult participants was 41 years; 80.6% were women, 87.7% were Latino, and median BMI was 34.9. The median age of children was 12 years; 50.9% were boys, 94.2% were Latino, and median BMI percentile for age was 94.2. Of the 103 children for whom we had data, 17 (16.5%) were overweight, 50 (48.5%) were obese, and 36 (35.0%) were normal weight. Of family types, Group 1 comprised 80 adults, Group 2 comprised 65 adults, and Group 3 comprised 97 adults and 106 children.

**Table 1:** Baseline Characteristics of Adult and Child Participants in the Y Living Program, a Family-Focused Lifestyle Intervention, San Antonio, 2012–2013^a^

Variables	Adults (n = 242)	Children (n = 106)
**Sex**		
Female	195 (80.6)	52 (49.1)
Male	47 (19.4)	54 (50.9)
**Age, median (IQR)**	41 (33–53)	12 (10–14)
**Ethnicity**		
Latino	171 (87.7)	82 (94.3)
Non-Latino	24 (12.3)	5 (5.7)
Missing data, n	47	19
**Race **		
African American	13 (7.0)	5 (5.7)
American Indian/Alaska Native	3 (1.6)	2 (2.3)
White	153 (82.3)	74 (85.1)
Asian/Native Hawaiian or other Pacific Islander	2 (1.1)	0
Other	15 (8.1)	6 (6.9)
Missing data, n	56	19
**Income, $**		
<5,000	17 (11.4)	4 (6.5)
5,000–9,999	10 (6.7)	7 (11.3)
10,000–14,999	25 (16.8)	6 (9.7)
15,000–24,999	29 (19.5)	11 (17.7)
25,000–34,999	36 (24.2)	14 (22.6)
35,000–49,999	28 (18.8)	18 (29.0)
≥50,000	4 (2.7)	2 (3.2)
Missing data, n	93	44
**Family structure**		
Group 1 (1 adult and no children)	80 (33.1)	0
Group 2 (>1 adult and no children)	65 (26.9)	0
Group 3 (≥1 adult and ≥1 child aged <18 y)	97 (40.1)	106 (100)
**Obesity status^b^**		
Normal	20 (8.4)	36 (35.0)
Overweight	43 (18.0)	17 (16.5)
Obese	176 (73.6)	50 (48.5)
Missing data, n	3	3
**Other weight-related measures, median (IQR)**		
BMI	34.9 (29.6–41.3)	—
zBMI	—	1.6 (0.4–2.3)
BMI percentile	—	94.2 (65.0–98.8)
Weight, lb	200.2 (163.6–237.3)	125.8 (90.0–157.8)
Waist circumference, in	41.0 (36.5–46.5)	30.8 (26.1–36.4)
% Body fat	42.7 (35.8–48.0)	31.0 (20.6–38.7)

Abbreviations: BMI, body mass index; IQR, interquartile range; zBMI, BMI *z* score.

a All values are number (percentage) unless otherwise indicated. Percentages in some categories may not sum to 100 because of rounding.

b For adults, normal if BMI < 25.0; overweight if 25.0 ≤ BMI <30.0; obese if BMI ≥ 30.0. For children, normal if BMI percentile < 85.0; overweight if 85.0 ≤ BMI percentile < 95.0; obese if BMI percentile ≥ 95.0.

Only 180 (74.4%) adults and 72 (67.9%) children completed the program; several baseline differences were found between those who completed the program and those who dropped out (Table 2). Compared with adults who completed the program, adults who dropped out were significantly younger (*P* = .04), more likely Latino (*P* = .03), and more likely in the group of adults and children (*P* < .001). Children who dropped out were significantly heavier at baseline than those who completed the program in all body composition measures (all *P* ≤ .04).

**Table 2:** Comparison of Participants Who Completed and Participants Who Dropped Out of the Y Living Program, a Family-Focused Lifestyle Intervention, San Antonio, 2012–2013^a^

Variable	Adults			Children		
	Completed (n = 180)[Table-fn T2FN2]	Dropped Out (n = 62)^b^	*P* Value^c^	Completed (n = 72)^b^^, ^ d	Dropped Out (n = 34)^b^	*P* Value^c^
**Sex**						
Female	148 (82.2)	47 (75.8)	.36	33 (45.8)	19 (55.9)	.45
Male	32 (17.8)	15 (24.2)		39 (54.2)	15 (44.1)	
**Age, median (IQR)**	43 (33–54)	38 (32–47)	.04	12 (10–13)	12 (10–14)	.52
**Ethnicity**						
Latino	121 (84.6)	50 (96.2)	.03	52 (94.6)	30 (93.8)	>.99
Non-Latino	22 (15.4)	2 (3.8)		3 (5.4)	2 (6.2)	
**Race**						
African American	11 (8.0)	2 (4.1)	.83	3 (5.4)	2 (6.2)	.37
American Indian/Alaska Native	2 (1.5)	1 (2.0)		2 (3.6)	0	
White	110 (80.3)	43 (87.8)		48 (87.3)	26 (81.2)	
Asian/Native Hawaiian or other Pacific Islander	2 (1.5)	0		0	0	
Other	12 (8.8)	3 (6.1)		2 (3.6)	4 (12.5)	
**Income, $**						
<5,000	14 (13.1)	3 (7.1)	.13	3 (7.9)	1 (4.2)	.92
5,000–9,999	4 (3.7)	6 (14.3)		4 (10.5)	3 (12.5)	
10,000–14,999	21 (19.6)	4 (9.5)		3 (7.9)	3 (12.5)	
15,000–24,999	18 (16.8)	11 (26.2)		6 (15.8)	5 (20.8)	
25,000–34,999	26 (24.3)	10 (23.8)		8 (21.0)	6 (25.0)	
35,000–49,999	20 (18.7)	8 (19.0)		12 (31.6)	6 (25.0)	
≥50,000	4 (3.7)	0 (0)		2 (5.3)	0	
**Family structure**						
Group 1 (1 adult and no children)	67 (37.2)	13 (21.0)	<.001	—	—	—
Group 2 (>1 adult and no children)	54 (30.0)	11 (17.7)		—	—	
Group 3 (≥1 adult and ≥1 child aged <18 y)	59 (32.8)	38 (61.3)		72 (100)	34 (100)	
**Obesity status^e^**						
Normal	14 (7.8)	6 (10.2)	.54	30 (42.2)	6 (18.8)	.04
Overweight	35 (19.4)	8 (13.6)		12 (16.9)	5 (15.6)	
Obese	131 (72.8)	45 (76.3)		29 (40.8)	21 (65.6)	
**Other weight-related measures, median (IQR)**						
BMI	34.8 (29.4–1.3)	35.1 (30.5–41.0)	.89	—	—	—
zBMI	—	—	—	1.4 (0.3–2.1)	1.9 (1.2–2.4)	.02
BMI percentile	—	—	—	91.1 (61.1–98.3)	97.1 (89.1–99.2)	.02
Weight, lb	200.2 (161.8–236.7)	198.9 (164.9–241.8)	.89	117.4 (79.4–154.2)	137.8 (117.6–159.5)	.02
Waist circumference, in	40.5 (36.4–46.0)	42.8 (37.8–48.1)	.22	28.5 (25.0–35.0)	33.4 (30.1–47.7)	.001
% Body fat	42.7 (35.6–48.2)	42.0 (37.0–46.4)	.43	28.7 (17.1–36.0)	34.9 (28.5–40.8)	.03

Abbreviations: BMI, body mass index; IQR, interquartile range; zBMI, BMI *z* score.

a All values are number (percentage) unless otherwise indicated. Percentages in some categories may not sum to 100 because of rounding.

b Not all categories sum to n in column because of missing values.

c *P* value based on Mann–Whitney *U* test or Fisher exact test.

d One child had no baseline measurements.

e For adults, normal if BMI < 25.0; overweight if 25.0 ≤ BMI <30.0; obese if BMI ≥ 30.0. For children, normal if BMI percentile < 85.0; overweight if 85.0 ≤ BMI percentile < 95.0; obese if BMI percentile ≥ 95.0.

Among adults who completed the program and had both baseline and postintervention measures (n ranged by measure from 173–180 adults), the absolute changes in BMI (median = −0.2; interquartile range [IQR], −1.0 to 0.3), weight (median = −1.6 lb; IQR, −5.5 to 1.2 lb), waist circumference (median = −1.0 in; IQR, −2.5 to 0 in) and percentage body fat (median = −0.6; IQR: −1.8 to 0.7) were significantly less than zero (all *P* < .001), indicating that on average the intervention significantly improved the body composition of adults (Table 3). The percentage changes in BMI, weight, waist circumference, and percentage body fat were also significantly less than zero (all *P* ≤ .001); on average these measures significantly decreased by 0.8% to 2.6% after the intervention. Among children who completed the program and had both baseline and postintervention measures (n = 71), the absolute changes in zBMI (median = 0.1; IQR, −0.01 to 0.2), BMI percentile (median = 0.4; IQR, −0.03 to 3.5), weight (median = 2.6 lb; IQR, 0 to 5.0 lb) and percentage body fat (median = 0.65; IQR, −1.12 to 2.68) were positive in sign (all *P* < .04), indicating that on average these body composition measures significantly increased postintervention. The percentage changes in BMI percentile, weight, and percentage body fat were also positive in sign (all *P* < .03), showing that on average these measures significantly increased by 0.4% to 2.7% after the intervention.

**Table 3:** Changes in BMI, Weight, Waist Circumference, and Percentage Body Fat Among Participants^a^ in the Y Living Program, a Family-Focused Lifestyle Intervention, San Antonio, 2012–2013

Measure	Adults			Children		
	n	Median (IQR)	*P* Value^b^	n^c^	Median (IQR)	*P* Value^b^
**Absolute change^d^**						
BMI	173	−0.2 (−1.0 to 0.3)	<.001	—	—	—
zBMI	—	—	—	71	0.1 (−0.01 to 0.2)	.001
BMI percentile	—	—	—	71	0.4 (−0.03 to 3.5)	.001
Weight, lb	180	−1.6 (−5.5 to 1.2)	<.001	71	2.6 (0 to 5)	<.001
Waist circumference, in	178	−1.0 (−2.5 to 0)	<.001	71	0 (−1.0 to 1.0)	.69
% Body fat	177	−0.6 (−1.8 to 0.7)	<.001	71	0.6 (−1.1 to 2.7)	.03
**% Change^e^**						
BMI	173	−0.8 (−2.6 to 0.9)	<.001	—	—	—
BMI percentile	—	—	—	71	0.4 (−0.03 to 6.8)	.001
Weight, lb	180	−0.8 (−2.7 to 0.7)	<.001	71	2.7 (0 to 4.6)	<.001
Waist circumference, in	178	−2.6 (−5.4 to 0)	<.001	71	0 (−4.0 to 2.6)	.67
% Body fat	177	−1.3 (−4.2 to 1.8)	.001	71	1.9 (−3.8 to 10.3)	.03

Abbreviations: BMI, body mass index; IQR, interquartile range, zBMI, BMI *z* score.

a Among participants who completed the program and had both baseline and postintervention measures.

b *P* value based on Wilcoxon signed-rank test.

c One child had no baseline measurements.

d Absolute change was calculated as week 12 measurement minus baseline measurement.

e % Change was calculated as (absolute change divided by baseline measure) multiplied by 100.

An examination of the association between baseline characteristics and change scores showed that the intervention effect varied across subgroups. Women had a greater decrease in BMI (*P* = .02) and weight (*P* = .047) than men. Compared with normal-weight adults, adults who were obese at baseline had a greater decrease in weight (*P* = .03), percentage change in BMI (*P* = .03), and percentage change in weight (*P* = .004); and those overweight at baseline had a significantly greater decrease in percentage change in weight (*P* = .02). Among children, girls had a greater increase in percentage body fat than boys (*P* = .03). Children in families with an annual household income of $15,000 or more at baseline had a smaller increase in percentage body fat (*P* = .004). Compared with normal-weight children, children who were obese at baseline had a smaller increase in percentage body fat (*P* = .001) and smaller percentage change in percentage body fat (*P* = .005).

## Discussion

Y Living was implemented and evaluated using a pragmatic approach (16). We observed improvement in body composition (measured by weight, waist circumference, and body fat) of adult participants who completed the 12-week program. Among child participants, all weight-related measures increased, as expected for this age group (17); however, improvements in weight and body fat were observed for children who were overweight or obese. Lack of a control group and wide variation of children’s ages makes the interpretation of children’s results difficult (17).

Generally, family-based studies have not examined intervention effects on adults when both adult and child family members were targeted by the intervention. We are the first to investigate the feasibility of a family-focused program to engage all members of low-income Latino households. Family structure (individual adult, multiple adults, or adults with children) did not affect weight change of adult program participants. As a weight-gain prevention program, our finding for adults is consistent with adult-only community-based weight gain prevention studies that led to successful weight maintenance without significant weight loss (18,19). Intervention activities were developed for a Latino, intergenerational, low-income, urban population. That this program was designed with participants’ cultural beliefs and local practices in mind may have contributed to positive weight change in adults (20). Latinos prefer health programs in a group setting that provide opportunities for sharing experiences and support among group members (21,22); participants enrolled as individuals may have benefited from the group support.

Other family-focused weight-gain prevention interventions similar to Y Living demonstrate that small sustainable lifestyle behavior changes are feasible and can prevent excess weight gain and promote healthy lifestyle adoption among participants (4–6). Although weight outcomes for children in our program were mixed, weight improvements among adults may portend weight improvements among children when parents adopt healthy behaviors and become healthy role models (14). Most adults in Y Living were women. We cannot say if this resulted from family structure (ie, more single-female–parent families), behavior (ie, fathers in 2-parent families do not tend to enroll) or attitudes and norms (ie, family health is traditionally a female domain). Latino mothers and fathers have important, yet distinctive, roles in ensuring their families adopt healthy lifestyles (23). In addition, because mothers and fathers play different roles in supporting children’s involvement in PA and sports, it is important to involve both parents (23). Although programs may benefit from including fathers, further research on parental roles in Latino families is needed to understand how to engage both parents.

Our study had several limitations. First, lack of a control group hinders our ability to accurately interpret results, in particular understanding effects on child outcomes, because we cannot control the increase in weight and body fat due to normal growth and environmental influences (24). Second, young adults and families with children had higher drop-out rates, indicating that the program may not have addressed some barriers to participation. Of the families with children, about one in 10 also had at least one young adult (18–24 y); these families had the highest dropout rate (71.4%). All participants received the same education content, of which some was less relevant to children or young adults and could have led to higher dropout rates. The structure of families in Y Living was diverse. Maintaining involvement in the program may require child–adult separation for some activities. More research is needed on how young adult family members can be better integrated into the program. Finally, Y Living program was offered as a voluntary, no-charge program to low-income community residents, which may have led to a bias in selection of participants.

## Appendix A. Y Living Program Session Topics and Objectives, San Antonio, 2012–2013

**Table:**

Week	Session	Topic	Objective
1	A	Join the Journey	Receive introduction to Y Living.
1	B	Got Purpose	Explore the connection between health, wellness, and spirituality.
2	A	Goal Setting and Journaling	Learn the basic principles of goal setting and self-monitoring.
2	B	Building a Support System	Identify members of their personal support system.
2	C	Wellness Consultation	Create food and activity plan (goal setting).
3	A	Jumpstarting an Active Lifestyle	Evaluate the health benefits of regular physical activity and the health consequences of inactivity.
3	B	1st Steps to Fitness	Review physical activity safety topics: injury prevention, hydration, environmental safety, clothing/shoes, gym etiquette.
4	A	Getting to Know My Plate	Review key My Plate messages, such as making half your plate fruits and vegetables, make half your grains whole, and switching to fat-free or low-fat milk.
4	B	Fiber, Fruits, and Veggies	Discuss strategies for increasing intake of fiber, fruits, and vegetables.
5	A	Label Reading	Identify a nutrition label’s components.
5	B	Meal Planning & Budgeting	Learn the 3 steps for healthy eating on a budget: planning, purchasing, and preparing.
5	C	Wellness Consultation	Check progress toward goals (compare goals to monitoring records) and write new behavior goals for upcoming week.
6	A	Finding Your Balance	Learn how healthy eating and being physically active affect calorie balance.
6	B	Super-Size Me	Learn how to make healthy selections when eating out.
7	A	Preparing Healthy Meals	Learn about small changes that can be made to prepare healthier foods with less fat, salt, and added sugars.
7	B	Healthy Snacks	Prepare 3 quick, budget-friendly, healthy snacks.
8	A	Rethink Your Drink	Discuss benefits of making healthier drinking choices.
8	B	The Lifestyle-Health Connection	Review the connection between overweight/obesity and health, including the increased risk of chronic disease.
9	A	Coping With Stress, Depression	Explore strategies for coping with stress and depression.
9	B	Boost Your Confidence	Identify strategies for increasing self-confidence.
9	C	Wellness Consultation	Check progress toward goals (compare goals to monitoring records) and write new behavior goals for upcoming week.
10	A	Financial Fitness	Learn about personal finance strategies that reduce stress and contribute to a healthy lifestyle.
10	B	Creating a Family Budget	Learn how to create and follow a family budget.
11	A	Small Steps for Lasting Change	Discuss ways to make physical activity fun and part of the daily routine.
11	B	Becoming a Health Ambassador	Learn about the Y Living Health Ambassador program.
12	A	Reflections	Learn to live a balanced life spiritually, physically, and emotionally.
12	B	Staying the Course	Reflect on wellness journey during the program and identify near- and long-term individual and family wellness goals.

## Appendix B. Distribution Analysis for Each Change Measure of Interest, Y Living Program, San Antonio, 2012–2013

This file is available as a downloadable Word document.
