# Supplementary material for: Weight Outcomes of Latino Adults and Children Participating in the Y Living Program, a Family-Focused Lifestyle Intervention, San Antonio, 2012–2013
Source: Prev Chronic Dis. 2015 Dec 10;12:E219. doi: 10.5888/pcd12.150219 (PMC4676278; doi:10.5888/pcd12.150219)
Supplement: Supplementary file 1 [file 15_0219_AppendixB.docx]

# Appendix B. Distribution Analysis for Each Change Measure of Interest, Weight Outcomes of Latino Adults and Children Participating in the Y Living Program, a Family-Focused Lifestyle Intervention, San Antonio, 2012–2013

| **Measures** | **Adult** | | | **Child** | | |
| --- | --- | --- | --- | --- | --- | --- |
|  | **n** | **Skewness^a^** | **Kurtosis^b^** | **n** | **Skewness^a^** | **Kurtosis^b^** |
| **Absolute change** | | | | | | |
| BMI | 173 | −1.41 | 13.91 | — | — | — |
| zBMI | — | — | — | 71 | 0.27 | 11.38 |
| BMI percentile | — | — | — | 71 | −0.99 | 12.71 |
| Weight | 180 | −0.64 | 5.3 | 71 | −0.87 | 9.30 |
| Waist circumference | 178 | −0.37 | 7.31 | 71 | −0.45 | 3.96 |
| % Body fat | 177 | −4.66 | 38.3 | 71 | 1.89 | 13.86 |
| **% Change** | | | | | | |
| BMI | 173 | 0.03 | 9.36 | — | — | — |
| BMI percentile | — | — | — | 71 | 2.26 | 12.46 |
| Weight | 180 | −0.18 | 4.88 | 71 | 1.76 | 13.98 |
| Waist Circumference | 178 | 0.33 | 6.07 | 71 | −0.12 | 3.91 |
| % Body fat | 177 | −1.22 | 30.87 | 71 | 3.73 | 17.89 |

^a^ Skewness is an indicator used in distribution analysis as a sign of asymmetry and deviation from a normal distribution.

- Skewness > 0: right-skewed distribution
- Skewness < 0: left-skewed distribution
- Skewness = 0: mean = median; the distribution is symmetrical around the mean.

^b^Kurtosisis an indicator used in distribution analysis as a sign of flattening or “peakedness” of a distribution.

- Kurtosis > 3: leptokurtic distribution, sharper than a normal distribution, with values concentrated around the mean and thicker tails. This means high probability for extreme values.
- Kurtosis < 3: platykurtic distribution, flatter than a normal distribution with a wider peak. The probability for extreme values is less than for a normal distribution, and the values are wider spread around the mean.
- Kurtosis = 3: mesokurtic distribution (normal distribution, for example).

# Skewness and Kurtosis Tests for Normality Among Adults and Children

| **Variable** | **n** | **Pr (Skewnesss)** | **Pr (Kurtosis)** | **Adjusted χ^2^** | **Prob > χ^2^** |
| --- | --- | --- | --- | --- | --- |
| **Adults** | | | | | |
| Change in BMI | 173 | 0.0000 | 0.0000 | 60.22 | 0.0000 |
| Change in weight | 180 | 0.0007 | 0.0002 | 20.15 | 0.0000 |
| Change in waist circumference | 178 | 0.0403 | 0.0000 | 22.61 | 0.0000 |
| Change in percentage body fat | 177 | 0.0000 | 0.0000 | —^a^ | 0.0000 |
| Percentage change in BMI | 173 | 0.8667 | 0.0000 | 25.08 | 0.0000 |
| Percentage change in weight | 180 | 0.3076 | 0.0009 | 10.56 | 0.0051 |
| Percentage change in waist circumference | 178 | 0.0676 | 0.0000 | 17.43 | 0.0002 |
| Percentage change in percentage body fat | 177 | 0.0000 | 0.0000 | 73.47 | 0.0000 |
| **Children** | | | | | |
| Change in BMIz | 71 | 0.3177 | 0.0000 | 18.07 | 0.0001 |
| Change in BMI percentile | 71 | 0.0012 | 0.0000 | 25.88 | 0.0000 |
| Change in weight | 71 | 0.0036 | 0.0000 | 20.48 | 0.0000 |
| Change in waist circumference | 71 | 0.1045 | 0.0862 | 5.40 | 0.0673 |
| Change in percentage body fat | 71 | 0.0000 | 0.0000 | 37.23 | 0.0000 |
| Percentage change in BMI percentile | 71 | 0.0000 | 0.0000 | 39.93 | 0.0000 |
| Percentage change in weight | 71 | 0.0000 | 0.0000 | 35.85 | 0.0000 |
| Percentage change in waist circumference | 71 | 0.6472 | 0.0952 | 3.11 | 0.2107 |
| Percentage change in percentage body fat | 71 | 0.0000 | 0.0000 | 58.63 | 0.0000 |

^a^ The adjusted χ^2^ cannot be calculated for this variable; the unadjusted χ^2^ of 211.56 indicated that the distribution is far from normal (*P* < .001).
